# Long-Term Impact of Severe Postoperative Complications after Esophagectomy for Cancer: Individual Patient Data Meta-Analysis

**DOI:** 10.3390/cancers16081468

**Published:** 2024-04-11

**Authors:** Davide Bona, Michele Manara, Gianluca Bonitta, Guglielmo Guerrazzi, Juxhin Guraj, Francesca Lombardo, Antonio Biondi, Marta Cavalli, Piero Giovanni Bruni, Giampiero Campanelli, Luigi Bonavina, Alberto Aiolfi

**Affiliations:** 1 I.R.C.C.S. Ospedale Galeazzi—Sant’Ambrogio, Division of General Surgery, Department of Biomedical Science for Health, University of Milan, 20157 Milan, Italy; davide.bona@unimi.it (D.B.); michele.mnra@gmail.com (M.M.); bbonit@icloud.com (G.B.); guerrazzi.gug@gmail.com (G.G.);; 2 Department of General Surgery and Medical Surgical Specialties, G. Rodolico Hospital, Surgical Division, University of Catania, 95131 Catania, Italy; abiondi@unict.it; 3 I.R.C.C.S. Ospedale Galeazzi—Sant’Ambrogio, Division of General Surgery, Department of Surgery, University of Insubria, 20157 Milan, Italy; 4 Department of Biomedical Sciences for Health, Division of General and Foregut Surgery, IRCCS Policlinico San Donato, University of Milan, 20097 Milan, Italy

**Keywords:** esophagectomy, esophageal cancer, severe postoperative complications, overall survival

## Abstract

**Simple Summary:**

After esophageal resection for cancer, severe postoperative complications (SPCs) have been reported in up to 20% of patients. These are associated with prolonged hospital stay, augmented costs, need for supplementary treatments, and increased 90-day mortality. The global effect of SPCs on long-term survival after esophagectomy is discussed, whereas, in the current literature, it is not frequently covered. The present systematic review and individual patient data meta-analysis suggests a statistically significant detrimental effect of SPCs on 5-year overall survival in patients undergoing curative esophagectomy for cancer. Also, a clinical trend toward reduced 5-year cancer specific survival and disease-free survival was perceived.

**Abstract:**

Background. Severe postoperative complications (SPCs) may occur after curative esophagectomy for cancer and are associated with prolonged hospital stay, augmented costs, and increased in-hospital mortality. However, the effect of SPCs on survival after esophagectomy is uncertain. Aim. To assess the impact of severe postoperative complications (SPCs) on long-term survival following curative esophagectomy for cancer, we conducted a systematic search of PubMed, MEDLINE, Scopus, and Web of Science databases up to December 2023. The included studies examined the relationship between SPCs and survival outcomes, defining SPCs as Clavien–Dindo grade > 3. The primary outcome measure was long-term overall survival (OS). We used restricted mean survival time difference (RMSTD) and 95% confidence intervals (CIs) to calculate pooled effect sizes. Additionally, we applied the GRADE methodology to evaluate the certainty of the evidence. Results. Ten studies (2181 patients) were included. SPCs were reported in 651 (29.8%) patients. The RMSTD overall survival analysis shows that at 60-month follow-up, patients experiencing SPCs lived for 8.6 months (95% Cis −12.5, −4.7; *p* < 0.001) less, on average, compared with no-SPC patients. No differences were found for 60-month follow-up disease-free survival (−4.6 months, 95% CIs −11.9, 1.9; *p* = 0.17) and cancer-specific survival (−6.8 months, 95% CIs −11.9, 1.7; *p* = 0.21). The GRADE certainty of this evidence ranged from low to very low. Conclusions. This study suggests a statistically significant detrimental effect of SPCs on OS in patients undergoing curative esophagectomy for cancer. Also, a clinical trend toward reduced CSS and DFS was perceived.

## 1. Introduction

Esophageal cancer is the sixth most common cause of neoplasm-related mortality worldwide, accounting for 5.6% of cancer-related mortality [1,2,3]. Surgery is the mainstay of treatment for both early and locally advanced resectable diseases [4,5,6]. Regardless of recent improvements in surgical techniques, preoperative risk assessment, and refined perioperative management, morbidity after esophagectomy remains substantial, even in high-volume centers [7,8,9]. Severe postoperative complications (SPCs) have been reported in up to 20% of patients and are associated with prolonged hospital stay, augmented costs, need for supplementary treatments, and increased 90-day mortality [10,11,12,13,14,15].

The impact of SPCs on survival after esophagectomy is still discussed because strong evidence is missing [16,17,18,19,20,21,22,23,24,25,26]. Hence, the purpose of this investigation was to assess the influence of SPCs on long-term survival after curative esophagectomy for cancer using a multivariate method for the meta-analysis of restricted mean survival time difference (RMSTD) with individual patient data (IPD).

## 2. Materials and Methods

A systematic review was reported according to the Preferred Reporting Items for Systematic Review and Meta-Analyses (PRISMA 2020) guidelines [27]. PubMed, Scopus, MEDLINE, Cochrane Central Library, Web of Science, and ClinicalTrials.gov were used [28] using the following Medical Subject Heading (MeSH) search terms: esophageal neoplasm, esophageal cancer, esophageal tumor, complication, postoperative compl*, Clavien–Dindo, survival, overall surv*, cancer-specific survival, and disease-free survival. Articles published from 1 January 2000 to 31 December 2023 were screened, together with the relevant references. A manual cross-reference search of the eligible papers was performed to identify additional important papers. This study is based on previously published studies and therefore did not require any additional ethical approval. The study was registered with PROSPERO (CRD42023453685).

### 2.1. Eligibility Criteria

Inclusions criteria: (a) studies reporting Kaplan–Meier curves comparing the effect of SPCs vs. no SPCs on survival in patients who underwent curative esophagectomy for cancer, (b) studies reporting data on overall survival (OS), cancer-specific survival (CSS), or disease-free survival (DFS). Exclusion criteria: (a) studies with mixed population survival data, (b) patients stratified according to complications classification other than Clavien–Dindo (CD), (c) editorials, (d) review articles, (e) case reports, and (f) studies involving fewer than 20 cases.

### 2.2. Selection Process

Three independent reviewers (MM, GG, and JG) conducted a literature review separately, adhering to the established inclusion criteria. Initial screening based on the title and abstract was carried out using the Rayyan Intelligent Systematic Review tool, followed by a full review of the eligible articles. Upon the removal of duplicates, any disagreements were resolved by two additional blinded reviewers (AA and DB).

### 2.3. Data Extraction

The reviewers (MM, GG, and JG) independently analyzed and recorded data using pro forma tables on Google Sheets, each filling out predetermined variables. These variables encompassed various aspects of the study, including author, publication year, country, inclusion/exclusion criteria, study design, population demographics (such as number, age, sex, body mass index, American Society of Anesthesiologists physical status), tumor characteristics (histology, location, neoadjuvant and adjuvant therapy), surgical treatment details (surgical approach, anastomotic technique, lymphadenectomy fields, pathologic tumor staging, and residual tumor classification), and postoperative complications outcomes according to the Clavien–Dindo (CD) classification. Kaplan–Meier curves related to the outcomes of interest were also collected alongside these data. Subsequently, two other authors (AA and GB) compared all the data at the conclusion of the review process to identify and resolve any discrepancies.

### 2.4. Outcomes and Definitions

The primary outcome of the study was overall survival (OS), with disease-free survival (DFS) and cancer-specific survival (CSS) considered as secondary outcomes. OS was defined as the duration from surgery to the last known follow-up and death from any cause. CSS was defined as the time from diagnosis to death specifically attributed to esophageal cancer. DFS was defined as the interval from surgical resection to local recurrence. Survival data were derived from Kaplan–Meier survival curves. Postoperative complications were graded according to the CD classification with the following grades: Grade 0 for no complications, Grade 1 for deviations from normal postoperative course without medical intervention, Grade 2 for complications requiring pharmacological treatment, Grade 3 for complications necessitating surgical, endoscopic, or radiological intervention (3a not requiring general anesthesia, 3b requiring general anesthesia), Grade 4 for life-threatening complications requiring intensive care unit management, and Grade 5 for death [29]. SPCs were defined as the presence of a CD complication graded more or equal to 3, whereas no SPCs were defined as CD ≤ 2 [21,22,26].

### 2.5. Quality Assessment and Assessment of Certainty of Evidence

The methodological quality of the included studies was independently assessed by three authors (MM, AA, and GB) using the ROBINS-I tool for observational studies [30]. This tool evaluates various domains, including confounding bias, selection bias, classification bias, intervention bias, missing data bias, outcomes measurement bias, and reporting bias, each categorized as a “Low”, “Moderate”, “Serious”, or “Critical” risk of bias. The overall judgment for each study falls into one of four categories: low, moderate, serious, or critical risk of bias. Furthermore, we utilized the Grading of Recommendations, Assessment, Development, and Evaluation (GRADE) tool to evaluate the quality of evidence across studies. GRADE evidence profiles were created for each comparison and outcome using GRADEpro (https://www.gradepro.org (accessed on 15 January 2024). The certainty of the evidence is determined by assessing the risk of bias across studies, as well as incoherence, indirectness, imprecision, publication bias, and other relevant parameters.

### 2.6. Statistical Analysis

The results of the systematic review were qualitatively summarized and synthesized into a frequentist meta-analysis of restricted mean survival time difference (RMSTD) based on several studies [31,32,33]. To accomplish this, individual patient time-to-event data were reconstructed from Kaplan–Meier curves following the method outlined by Guyot [34]. The Kaplan–Meier curves were digitized using specialized software (Get Data Graph Digitizer v2.26). The computation of the RMSTD involved a random effect multivariate meta-analysis that leveraged strength across different time points, with a within-trial covariance matrix derived via bootstrapping (1000 iterations) and a restriction time of 60 months. Additionally, utilizing individual patient data (IPD), a flexible hazard-based regression model was employed, incorporating a normally distributed random intercept. In this model, the baseline hazard was represented by the exponential of a B-spline of degree 3 with no interior knots, and model selection was guided by the Akaike Information Criterion (AIC). The time-dependent effects of surgical treatment were parameterized as interaction terms between surgical treatment and baseline hazard, with statistical significance assessed using the likelihood ratio test. Two-sided p values were deemed statistically significant when below 0.05, and confidence intervals were computed at the 95% level. All analyses were conducted using the R software application (version 3.2.2; R Foundation, Vienna, Austria) [35,36].

## 3. Results

### 3.1. Systematic Review

**Figure 1:**
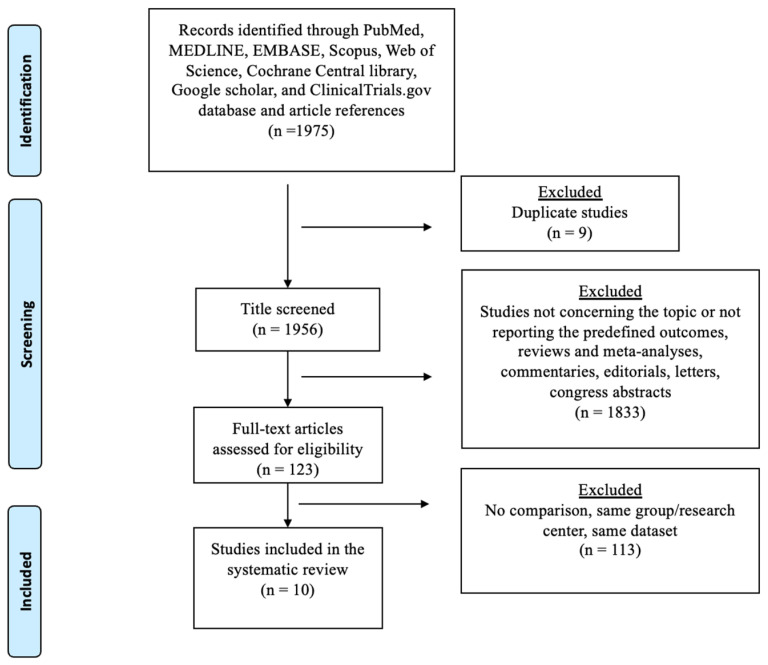
The PRISMA (Preferred Reporting Items for Systematic Reviews checklist (File S1) diagram.

The selection process flowchart, depicted in Figure 1, illustrates the study selection. Initially, 1975 publications were screened following the removal of duplicates, resulting in 123 articles undergoing full-text review. Following thorough evaluation, eleven observational papers satisfied both the inclusion and exclusion criteria and were subsequently included in the quantitative analysis. The quality of the included studies is listed in Supplementary Table S1.

Overall, 2181 patients undergoing esophagectomy for cancer were incorporated for quantitative synthesis (Table 1). SPCs were reported in 651 (29.8%) patients. The patients’ age ranged from 32 to 86, the majority were males (83.5%), preoperative BMI ranged from 18 to 38, and 14% of subjects were ASA score > 3. Squamous cell carcinoma (51.8%) and adenocarcinoma (46.3%) were the most frequently reported tumor histology. Tumor location was reported in seven studies (1493 patients) and distributed in the distal esophagus/esophagogastric junction (68.9%), thoracic (25.1%), and cervical (6%) esophagus [16,17,19,20,22,23,24]. Neoadjuvant chemoradiation treatment was performed in 75% of patients with different protocols and regimens. Minimally invasive, hybrid, and open Ivor Lewis or McKeown esophagectomy were reported. Two-field and three-field lymphadenectomy were described, whereas anastomotic techniques were heterogeneous among the included studies, mainly depending on operating surgeon preferences. Pathological tumor stage, as classified by the sixth, seventh, and eighth editions of the *American Joint Committee on Cancer*, was reported in five studies encompassing 1129 patients. The distribution of tumor stages was as follows: stage 0–I accounted for 25%, stage II for 29%, stage III for 41%, and stage IV for 5% of the patients [16,20,21,23,26].

### 3.2. Meta-Analysis—Overall Survival (OS)

**Figure 2:**
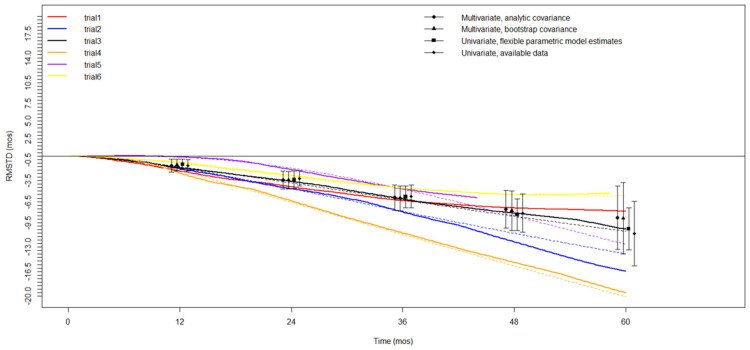
Restricted mean survival time difference (RMSTD) for OS. Each color represents a single study. The pooled RMSTD with relative 95% CIs using different statistical methods estimation is represented by black points (triangle, circle). Figure legend: X-axis represents the time in months (mos); Y-axis represents the RMSTD analysis for each included study.

**Figure 3:**
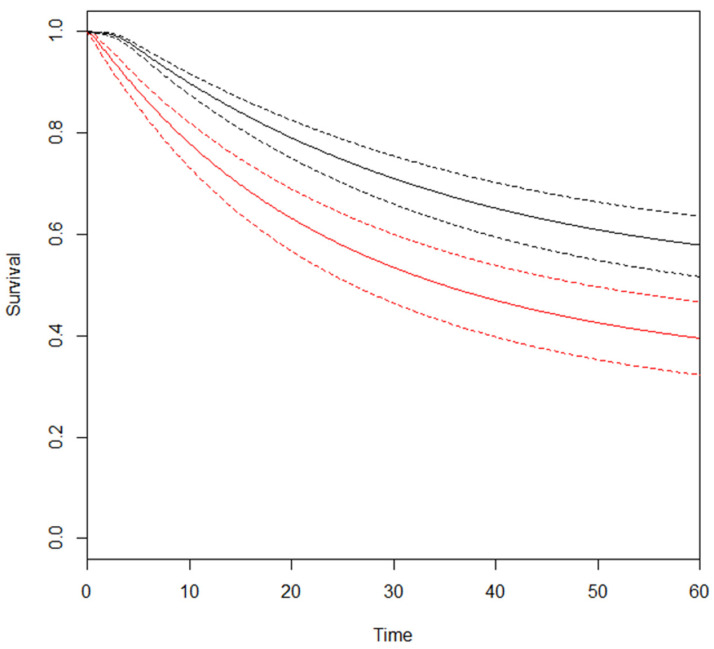
Estimated pooled OS (Y-axis) for SPC (red line) and no-SPC (black line) patients. Time (X-axis) is expressed in months.

The RMSTD clinical appraisal was estimated from six studies with a 5-year minimum follow-up [16,20,22,23,24,25]. Table 2 and the graphical representation (Figure 2) display the restricted mean survival time difference (RMSTD) and time horizons for overall survival (OS). The multivariate meta-analysis revealed an RMSTD estimate of −1.4 months at 12 months (95% CIs −2.4, −0.4), indicating that patients experiencing SPCs live 1.4 months less than those not experiencing SPCs. At 24-month follow-up (τ2), the combined effect from the RMSTD estimate is −3.4 months (95% CIs −4.6, −2.1). At 36-month follow-up (τ3), the combined effect from the multivariate meta-analysis with analytically derived covariance indicates an RMSTD of −5.8 months (95% CIs −7.7, −3.9). Similarly, at 48-month follow-up (τ4), the combined effect is −7.4 months (95% CIs −10.1, −4.7), and at 60-month follow-up (τ5), the combined effect is −8.6 months (95% CIs −12.5, −4.7). Figure 3 illustrates the estimated pooled overall survival (OS) for patients with and without SPCs.

### 3.3. Secondary Outcomes—CSS/DFS

The clinical DFS and CSS evaluation of the RMSTD was based on six and three studies, respectively [16,17,19,21,22,23,24,26]. The estimation of cancer-specific survival (CSS) at different time horizons is detailed in Table 3. At τ5 = 60-month, the multivariate meta-analysis yields a combined estimate of −6.8 months (95% CIs from −11.9 to 1.7), suggesting that SPCs do not appear to have a significant effect on long-term CSS. Similarly, the estimation of disease-free survival (DFS) at different time horizons is detailed in Table 4. At τ5 = 60-month, the multivariate meta-analysis provides a combined estimate of −4.6 months (95% CIs from −11.9 to 1.9), indicating that SPCs also do not seem to have a substantial effect on long-term DFS. The estimated pooled CSS and DFS curves for patients with no SPCs and SPCs are depicted in Supplementary Figures S1 and S2. Using the GRADE tool, the certainty of the evidence for the assessed outcomes was rated between very low and low due to concerns regarding confounding bias, inconsistency, and imprecision (Supplementary Table S2).

## 4. Discussion

This study shows that 5-year OS was reduced by 8.6 months on average in patients experiencing SPCs after esophagectomy, whereas a clinical trend toward worse CSS and DFS was noticed.

Complications following esophageal cancer surgery remain a major concern, with over 50% of patients experiencing them [37]. The most commonly reported complications include leakages at the anastomosis, heart rhythm anomalies, pneumonia/aspiration, chylothorax, and recurrent nerve damage [15,38]. Various factors contribute to these complications, including age, health conditions, nutritional status, prior chemoradiation therapy, surgical technique, and extent of lymph node dissection [39]. Timely identification and treatment of these complications are crucial for improving patient outcomes and their quality of life [40,41]. Managing these complications typically involves a collaborative effort among surgeons, gastroenterologists, oncologists, nutritionists, and physical therapists [42]. Close monitoring after surgery, including clinical assessments, lab tests (i.e., C-reactive protein), and imaging, aids in early detection and intervention [14]. These complications significantly impact recovery and quality of life, leading to physical symptoms like pain, swallowing difficulties, and fatigue, as well as psychological challenges such as anxiety and depression [43]. Further, they may prolong hospital stays, necessitate further treatments, and increase healthcare expenses, putting a strain on both patients and healthcare systems [44,45]. In these patients, adequate postoperative nutritional support is crucial for promoting wound healing, immune function, and overall recovery. This might involve supplements, feeding tubes, or dietary guidance to address malnutrition and support healing. Physical therapy and rehabilitation are integral for restoring function and quality of life post-esophagectomy. Finally, rehabilitation programs focusing on respiratory exercises and mobility aid in avoiding muscle wasting, thus regaining strength and independence [46]. We categorized postoperative complications according to the Clavien–Dindo classification, defining SPCs as those with a grade ≥3. Consistent with previous findings, our study estimated an incidence of SPCs at 29%. SPCs are associated with even worse short-term outcomes, longer hospital stays, increased costs, higher reoperation rates, and diminished quality of life [47].

The survival percentages after esophagectomy for cancer can vary depending on several factors, including age, general comorbidities, the stage of cancer at the time of surgery, smoke status, active alcohol consumption, histological type, tumor grading, tumor length >2 cm at the time of diagnosis, extent of lymphadenectomy, poor pathological response, intraoperative R1, postoperative transfusion requirements, hospital volumes, and experience of the operating surgeon [48,49,50,51,52,53,54,55,56]. Previously, the 5-year survival rate following esophagectomy has typically been reported to be between 15% and 25% [57]. However, in the last three decades, survival rates have progressively improved because enhanced of screening programs with early cancer detection, advancements in surgical techniques (i.e., minimally invasive approaches), the implementation of prehabilitation and enhanced recovery after surgery pathways, neoadjuvant and/or adjuvant chemoradiation treatments in patients with locally advanced disease, treatments focused on human epidermal growth factor 2 (HER-2) protein overexpression, gene amplification, and adjuvant immunotherapy protocols in PD-L1-positive patients [58,59,60,61,62,63,64,65].

The impact of postoperative complications and, in particular, of SPCs on long-term oncological outcomes has been discussed for a long time, but this impact still remains debated [7,8,9,66]. In a retrospective analysis conducted in 2004 at Memorial Sloan Kettering Cancer Center, involving 510 patients who underwent esophageal resection, those without technical complications showed better overall survival compared to those with such complications [67]. Similarly, a 2009 study at Leuven University Hospital found a significant correlation between complication severity and time to tumor recurrence among 150 patients who underwent transthoracic esophagectomy [18]. Another retrospective analysis in 2008 by Lagarde et al. concluded that postoperative complications independently correlate with a shorter interval to death from recurrence among 191 patients who succumbed to tumor recurrence [68]. Conversely, a 2006 study involving 522 patients undergoing thoracic esophagus and GE carcinoma resection suggested that long-term prognosis primarily hinges on tumor characteristics and is unaffected by surgical complications [69]. Similarly, a retrospective 2006 analysis performed at the University of Hong Kong Medical Centre involving 434 patients undergoing esophagectomy for squamous cell carcinoma reported no impact on long-term survival among patients with surgical complications [70]. Further, a retrospective 2020 review from the UK including 1100 patients undergoing esophagectomy or gastrectomy concluded that SPCs reduce median OS and DFS [71]. Another prospective Swedish database study of 567 patients demonstrated that the adjusted hazard ratio of mortality was modestly increased in patients who had a surgical complication, and therefore may be an independent predictor of long-term survival [72]. Finally, Fransen et al. showed in a 915-patient retrospective multicenter study that overall complications after minimally invasive esophagectomy seem to not be associated with a detrimental impact on overall long-term survival [47].

In our study, we observed that SPCs seem to be associated with a reduced 5-year life-expectancy of 8 months compared to patients not experiencing SPCs. This is similar to what has been previously reported by Luc et al., who defined reduced 5-year OS in patients suffering from SPCs after esophagectomy (*p* = 0.006) [22]. Similarly, Kiyozumi et al. and Bundred et al. observed significantly reduced long-term OS rates in patients experiencing SPCs [16,24]. In contrast, Yamamoto et al. reported no statistically significant survival difference in the long-term (50% vs. 49%) [21]. Different hypotheses could possibly explain this finding. Complications may lead to physiological stress, the activation of patients’ immune system, and systemic inflammation with the production of proinflammatory cytokines such as interleukin (IL)-6 and IL-8. This mechanism has been theorized to reduce the immune system’s capacity to suppress tumor recurrence. Additionally, inflammatory pathways have been shown to facilitate micro-metastases tumor growth with increased risk for local and distant metastases [73,74,75,76,77,78,79]. These mechanisms may be further amplified by transient immunodepression induced by additional invasive procedure sometimes required to treat SPCs [80,81,82,83]. Second, the decline in overall patient health caused by SPCs may lead to delays or reduced tolerance to adjuvant treatments. Interestingly, a recently published study reported that less than 30% of patients eligible for adjuvant treatment that experienced SPCs effectively received it [84]. Third, the onset of SPCs in patients with comorbidities may increase the risk of postoperative non-cancer-related mortality [85]. Notably, no statistically significant differences were detected in DFS and CSS up to 60-month follow-up. This is similar to Li et al., who did not show a significant effect of SPCs on 5-year CSS survival (43.2% vs. 43.5%) after minimally invasive esophagectomy [19]. Similarly, D’annoville et al. (*p* = 0.354) did not report significant long-term DFS differences [17]. In contrast, Luc et al. and Yamashita et al. described significantly reduced 5-year DFS (*p* = 0.045) and CCS (*p* = 0.016) in patients experiencing SPCs, respectively [22,26]. Despite the lack of statistical significance, a clinical trend toward reduced CSS (−6.8 months) and DFS (−4.6 months) was noticed in patients experiencing SPCs. The lack of statistical significance may be the effect of a statistical incongruity or may be correlated to the small number of studies analyzing data for DFS and CSS. Therefore, since the exclusive interpretation of OS data may be misleading because of the inclusion of non-cancer deaths, future studies should predominantly explore the effect of SPCs on CSS and DFS.

The GRADE certainty of this evidence ranged from low to very low; therefore, some concerns should be pondered while inferring our results. First, the variability in the reporting postoperative complications according to the Clavien–Dindo classification was high. Therefore, reporting bias and interstudy heterogeneity should be considered. The utilization of a generalized postoperative complication grading system, such as the Clavien–Dindo, may dilute the real impact of specific complications such as anastomotic leak and pulmonary complication. Therefore, focusing on definite complications rather than a generalized classification system could provide more useful insights. Notably, two recent meta-analyses defined the negative impact of both anastomotic leak and pulmonary complications after esophagectomy [10,13]. Specifically, Aiolfi et al. suggest a clinical impact of AL on long-term OS after esophagectomy, with significantly reduced 60-month OS in patients experiencing postoperative anastomotic leak (−4.2 months; *p* < 0.001). Similarly, Manara et al. analyzed the effect of postoperative pulmonary complications and showed that patients not experiencing PC live for an average of 8.5 (95% CIs 6.2–10.8; *p* < 0.001) months longer compared with those with PC at 60-month follow-up. Also, patients not experiencing postoperative PC seem to have significantly longer CSS (8 months; *p* < 0.001) and DFS (5.4 months; *p* = 0.005). Second, all studies were from tertiary-level centers; the management of postoperative complications in high-volume centers with multidisciplinary facilities has been shown to be more effective through early recognition/treatment and consequent reduction in “failure to rescue” rates. Therefore, timely and effective postoperative complication management could mitigate the effects of SPCs on survival [41,86,87]. Third, the level of immune system compromise with micro-metastasis, neutrophils traps, and disease recurrence facilitation might also depend on the cumulative effect of multiple complications and duration with the persistent activation of the septic/inflammatory status [79]. Fourth, neoadjuvant and adjuvant therapies changed over the study period; therefore, this potential additional bias should be measured [60,88].

The principal strength of the present IPD analysis is the appraisal of long-term survival using the RMSTD methodology. RMSTD is gaining increasing consensus in clinical oncology as it is a robust and interpretable tool for assessing survival benefit, thus allowing, in the present analysis, for an estimation of the SPCs effect during follow-up. It matches the area under the Kaplan–Meier survival curves and is easier to understand compared to RR and HR, which may be misinterpreted because both suppose a constant risk during follow-up. We acknowledge that our study does have some limitations related to baseline heterogeneity (i.e., patient demographics, comorbidities, etc.) the and non-uniform reporting of oncologic data (i.e., histology, staging, grading, adjuvant treatments compliance, heterogeneous multidisciplinary perioperative care teams, or enhanced recovery after surgery programs). Our results should not be generalized because the sample was principally from Eastern centers, with a possible impact of tumor epidemiology and genomic characterization [89]. The different surgical approaches (i.e., open, hybrid, totally minimally invasive esophagectomy) and operating surgeon expertise/learning curve may have an effect on the incidence of SPCs [90,91]. Unfortunately, all the included studies graded postoperative complications based on the Clavien-Dindo classification, which is not the current standard for assessing esophagectomy-related morbidity and mortality. The contemporary benchmark, introduced in 2015 by the Esophageal Complications Consensus Group (ECCG), is now mostly accepted [15,92].

## 5. Conclusions

Our study suggests a statistically significant impact of SPCs on OS in patients undergoing curative esophagectomy for cancer. Also, a clinical trend toward reduced CSS and DFS was perceived. Since the existing evidence is not conclusive, robust and well-designed prospective studies are needed to further explore the effect of SPCs on CSS and DFS.

## Figures and Tables

**Table 1:** Summary of the demographic, clinical, and operative data for patients undergoing curative esophagectomy for cancer: Ret: retrospective; yrs: years; SCC: squamous cell carcinoma; ADK: adenocarcinoma; U: upper esophagus; M: medium esophagus; L: lower esophagus; pStage: pathologic tumor stage, reported according to the 6th, 7th, and 8th editions of the *American Joint Committee on Cancer* (AJCC); Hyb: hybrid esophagectomy; MIE: minimally invasive esophagectomy; nr: not reported The data are presented as numbers, mean ± standard deviation, and median (range).

Author, Year	Country	Study Design	No. Pts	Sex M	Age (yrs)	Tumor Histology (SCC-ADK-Other)	Location (U-M-L)	Neoadjuvant Treatment	pStage 0–I	pStage II	pStage III	pStage IV	Surgical Approach
D’annoville et al., 2012 [17]	France	Ret	341	286	60.1 ± 10	127-214-0	14-77-250	179	nr	nr	nr	nr	Open
Xia et al., 2013 [20]	USA	Ret	237	195	62 (32–86)	36-201-0	5-17-215	155	90	84	52	11	Open/MIE
Luc et al., 2015 [22]	France	Ret	116	106	64.6 (40–79)	0-106-0	0-0-116	106	nr	nr	nr	nr	Open
Yamashita et al., 2016 [26]	Japan	Ret	255	220	65 (35–85)	255-0-0	nr	255	49	76	120	10	Open/Hybrid
Aahlin et al., 2016 [25]	Norway	Ret	331	nr	nr	nr	nr	nr	nr	nr	nr	nr	nr
Li et al., 2017 [19]	China	Ret	214	170	60.2 ± 8.1	214-0-0	41-152-21	nr	nr	nr	nr	nr	Hybrid/MIE
Kiyozumi et al., 2018 [24]	Japan	Ret	50	46	nr	50-0-0	8-40-2	50	nr	nr	nr	nr	Open
Bundred et al., 2020 [16]	UK	Ret	430	342	64.9 ± 9.4	70-337-23	0-24-368	nr	89	95	232	9	Open/Hybrid/MIE
Yamamoto et al., 2020 [21]	Japan	Ret	102	92	nr	102-0-0	nr	42	28	30	31	13	Open/Hybrid
Kurokawa et al., 2020 [23]	Japan	Ret	105	88	63.6 ± 7.8	105-0-0	20-52-33	105	20	39	27	5	Open/Hybrid

**Table 2:** The restricted mean survival time difference (RMSTD) for overall survival at different time horizons for SPCs vs. no SPCs. SE—standard error; 95% Cis—confidence intervals; mos—months.

Time Horizon	No. Trials	RMSTD (mos)	SE	95% CIs	*p* Value
12-month	6	−1.4	0.5	−2.4, −0.4	0.008
24-month	6	−3.4	0.6	−4.6, −2.1	<0.001
36-month	5	−5.8	0.9	−7.7, −3.9	<0.001
48-month	5	−7.4	1.4	−10.1, −4.7	<0.001
60-month	3	−8.6	1.9	−12.5, −4.7	<0.001

**Table 3:** The restricted mean survival time difference (RMSTD) for cancer-specific survival at different time horizons for SPCs vs. no SPCs. SE—standard error; 95% Cis—confidence intervals; mos—months.

Time Horizon	No. Trials	RMSTD (mos)	SE	95% CIs	*p* Value
12-month	3	−0.7	0.3	−1.2, −0.2	0.009
24-month	3	−1.9	1.1	−4.0, 0.3	0.09
36-month	3	−3.3	1.9	−7.0, 0.4	0.08
48-month	3	−3.9	3.1	−10.1, 2.3	0.22
60-month	2	−6.8	2.6	−11.9, 1.7	0.21

**Table 4:** The restricted mean survival time difference (RMSTD) for disease-free survival at different time horizons for SPCs vs. no SPCs. SE—standard error; 95% Cis—confidence intervals; mos—months.

Time Horizon	No. Trials	RMSTD (mos)	SE	95% CIs	*p* Value
12-month	6	−0.6	0.4	−1.4, 0.2	0.13
24-month	6	−2.3	0.9	−4.2, −0.5	0.01
36-month	6	−3.7	1.6	−6.8, −0.5	0.02
48-month	6	−4.4	2.4	−9.2, 0.3	0.06
60-month	3	−4.6	3.3	−11.1, 1.9	0.16

## Data Availability

Data generated at a central, large-scale facility are available upon request from the corresponding author.

## Supplementary Materials

The following supporting information can be downloaded at: https://www.mdpi.com/article/10.3390/cancers16081468/s1, Figure S1. Estimated pooled CSS (Y-axis) for SPC (red line) and no-SPC (black line) patients. Time (X-axis) is expressed in months. Figure S2. Estimated pooled DFS (Y-axis) for SPC (red line) and no-SPC (black line) patients. Time (X-axis) is expressed in months. Table S1. Quality assessment of the included studies (ROBINS-I tool). Each domain is evaluated with one of the following: low, moderate, serious, and critical. The categories of judgement for each study are low, moderate, serious, and critical risk of bias. Table S2. The GRADE certainty of evidence. File S1: Systematic Reviews checklist.
