# Supplementary material for: Long-Term Impact of Severe Postoperative Complications after Esophagectomy for Cancer: Individual Patient Data Meta-Analysis
Source: Cancers (Basel). 2024 Apr 11;16(8):1468. doi: 10.3390/cancers16081468 (PMC11048031; doi:10.3390/cancers16081468)
Supplement: Supplementary file 1 [file cancers-16-01468-s001.zip › Table S1.pdf]

Study	Confounding Bias	Selection Bias	Classification Bias	Intervention Bias	Missing Data Bias	Measurement Bias	Reporting Bias	Bias
D'Annoville et al, 2012 [17]	Serious	Serious	Moderate	Moderate	Serious	Moderate	Serious	Serious
Xia et al, 2013 [20]	Moderate	Moderate	Serious	Moderate	Serious	Moderate	Serious	Serious
Luc et al, 2015 [22]	Moderate	Serious	Moderate	Serious	Moderate	Moderate	Moderate	Serious
Yamashita et al, 2016 [26]	Moderate	Serious	Moderate	Serious	Serious	Moderate	Moderate	Serious
Aahlin et al, 2016 [25]	Serious	Moderate	Moderate	Moderate	Serious	Serious	Serious	Serious
Li et al, 2016 [19]	Moderate	Serious	Moderate	Moderate	Serious	Moderate	Moderate	Serious
Kiyozumi et al, 2018 [24]	Serious	Serious	Moderate	Serious	Moderate	Moderate	Moderate	Serious
Bundred et al, 2020 [16]	Moderate	Moderate	Moderate	Moderate	Serious	Moderate	Moderate	Serious
Yamamoto et al, 2020 [21]	Serious	Serious	Serious	Moderate	Serious	Moderate	Moderate	Serious
Kurokawa et al, 2020 [23]	Serious	Serious	Serious	Serious	Moderate	Moderate	Moderate	Serious

Supplementary Table S1. Quality assessment of the included studies (ROBINS-I tool). Each domain is evaluated with one of the following: Low, Moderate, Serious, and Critical. The categories of judgement for each study are low, moderate, serious, and critical risk of bias.
