# Supplementary material for: Long-Term Impact of Severe Postoperative Complications after Esophagectomy for Cancer: Individual Patient Data Meta-Analysis
Source: Cancers (Basel). 2024 Apr 11;16(8):1468. doi: 10.3390/cancers16081468 (PMC11048031; doi:10.3390/cancers16081468)
Supplement: Supplementary file 1 [file cancers-16-01468-s001.zip › Table S2.pdf]

Author(s):
Question: SPC compared to no SPC for [Problema di salute e/o di popolazione]
Setting: Esophagectomy
Bibliography:

Certainty assessment							№ of patients		Effect		Certainty	Importance
**№ of studies**	**Study design**	**Risk of bias**	**Inconsistency**	**Indirectness**	**Imprecision**	**Other considerations**	**SPC**	**no SPC**	**Relative (95% CI)**	**Absolute (95% CI)**		
**Overall Survival**												
6	observational studies	serious	serious	not serious	serious	publication bias strongly suspected strong association all plausible residual confounding would reduce the demonstrated effect			-	median **8.6 lower** (12.5 lower to 4.7 lower)	⊕⊕◯◯ Low	
**Cancer Specific Survival**												
3	observational studies	serious	not serious	serious	very serious	publication bias strongly suspected strong association all plausible residual confounding would reduce the demonstrated effect			-	median **6.8 lower** (11.9 lower to 1.7 lower)	⊕◯◯◯ Very low	
**Disease-Free Survival**												
6	observational studies	serious	serious	not serious	very serious	publication bias strongly suspected strong association all plausible residual confounding would reduce the demonstrated effect			-	median **4.6 lower** (11.1 lower to 1.9 higher)	⊕◯◯◯ Very low	

CI: confidence interval
