# Supplementary figures and images for: Long-Term Impact of Severe Postoperative Complications after Esophagectomy for Cancer: Individual Patient Data Meta-Analysis
Source: Cancers (Basel). 2024 Apr 11;16(8):1468. doi: 10.3390/cancers16081468 (PMC11048031; doi:10.3390/cancers16081468)

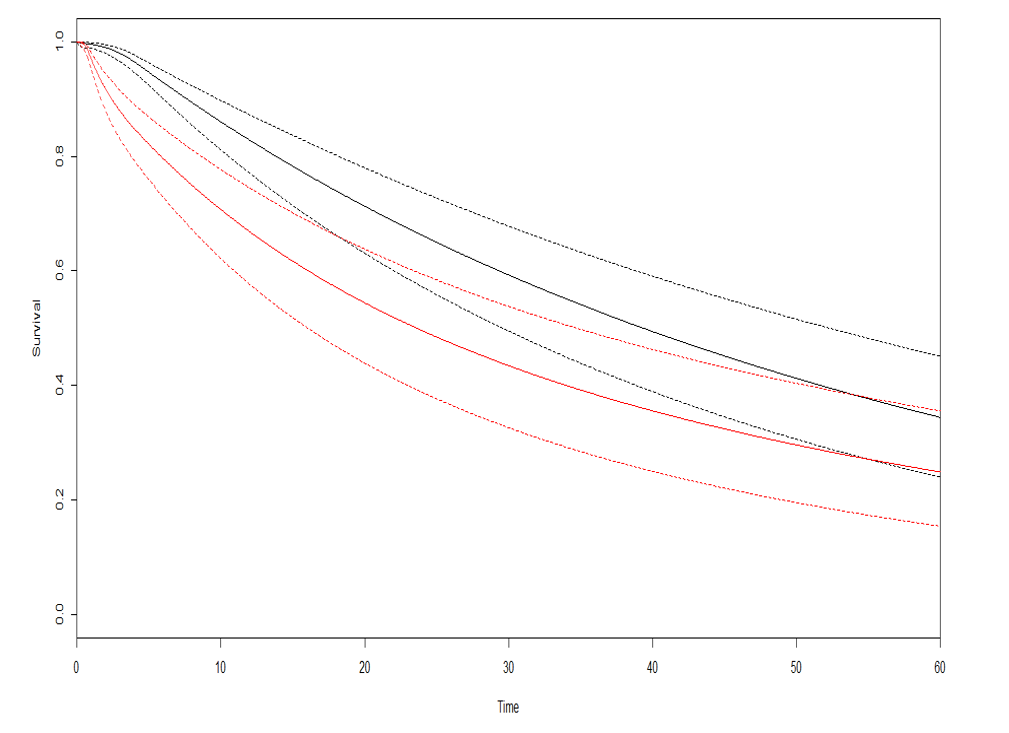

Supplement: Supplementary file 1 [file cancers-16-01468-s001.zip › Figure S1.tiff]

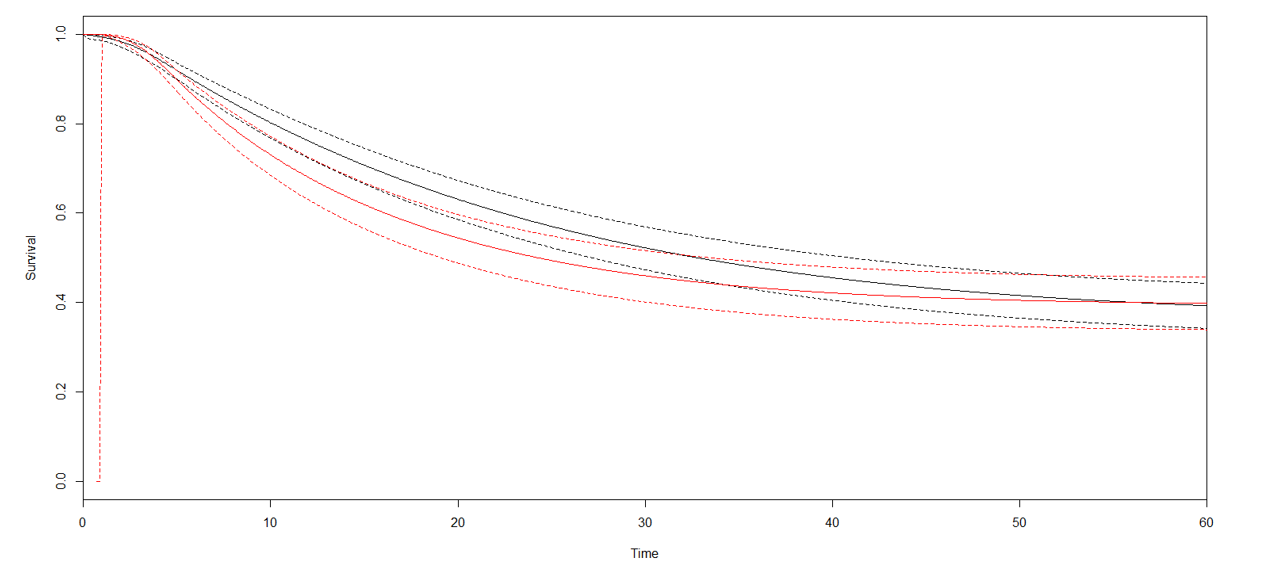

Supplement: Supplementary file 1 [file cancers-16-01468-s001.zip › Figure S2.tiff]
